# Remarks on the Medical Topography of Texas, and on the Diseases of the Army of Invasion

**Published:** 1847-05

**Authors:** George Johnson

**Affiliations:** Late Surgeon in the United States Army


*Remarks on the Medical Topography of Texas, and on the diseases of the army of invasion.* By GEORGE JOHNSON, M. D., late Surgeon in the United States Army.—The Brazos Santiago Island, Texas, has become a place of much importance since the present war with Mexico commenced. From May last to the present time, all troops, destined for the "army of occupation and invasion," have been landed at the Brazos, and on account of the difficulties of transportation at the commencement of the war, many of the regiments remained encamped upon the island for several weeks. During the last summer, most of the volunteer regiments have been stationed along the banks of the Rio Grande, between Matamoras and the mouth of the river. Much has been said in the newspapers, and elsewhere, of the unhealthfulness of this region, but I have not seen the true causes assigned for the great mortality which has occurred amongst our troops upon the Rio Grande; I will, therefore, at your request, give you a brief sketch of the medical topograpy of this region, together with some of the causes which have led to this mortality. Hereafter I hope to see this subject discussed by the medical officers of the army, many of whom have had far better opportunities than fell to my lot for obtaining correct information on this head, particularly those accomplished surgeons, Drs. Wood and Wells, at Point Isabel, and Dr. Wright and his assistants, at the general hospital at Matamoras.

The Brazos Island, it might be inferred from the many statements that have been made, is particularly unhealthy, from its location. This, I think, cannot be the case. Though a dreary and uninteresting sand bar, I believe it to be as healthy as Galveston, or any other spot along the Gulf coast. The island is about four miles in length, and one and a half in breadth. It would be almost level with the Gulf, but for the sand hills which line its southern extremity for half a mile. There are some two or three ponds on the island, called on the maps of the country, fresh water ponds, though I found them quite salty on trial. These ponds are situated about a mile and a half from the sand hills, (the place of encampment of the troops,) and to the north. The sea breeze blows almost continually from the south-west, so that no deleterious effects can arise from them. This breeze usually commences about 9 A. M. and continues throughout the night, making sleep delightful and refreshing. By it the sun's heat is rendered less oppressive, and there was not a day so warm, during our stay upon the Island, which was during the month of June, as to prevent the men of our regiment from perambulating it from end to end, on fishing and hunting excursions. Here, too, the men enjoyed the pleasure and benefit of bathing. I have understood that the Mexicans considered the Brazos healthy, prior to the arrival of our troops, and I learned from an American woman, a native of North Carolina, who has lived upon the Island for several years, that her family, consisting of six children, had enjoyed excellent health since her residence there. Yet she had occupied, during the whole time, a miserable little shed, only partially covered with ox hides.

The Mexicans who were taken prisoners at Palo Alto and Resaca de la Palma, were employed in the Quartermaster's department, at the Brazos, and though these men were exposed, day after day, to the heat of the sun, in their labours about the shipping, yet I never knew a case of sickness to occur amongst them. The other employees of the Quartermaster's department, such as teamsters, carpenters, etc., also retained their health, whilst the troops at the same period, were suffering with diarrhœas and dysenteries. I accounted for this circumstance, thus: these teamsters were making daily trips to the mouth of the Rio Grande, (nine miles,) and they kept their messes well supplied with the excellent water of that river. Besides, they had learned to cook their food properly, and they slept in the dry and comfortable Government storehouses, whilst the troops were lying under tents, upon the wet sand, eating food that was only partially cooked, and drinking the brackish water from the wells that it was almost impossible to retain upon the stomach.

It is known to all military men, and to the profession, that dysenteries and diarrhœas are camp diseases, and are common to every location where troops are encamped for a few weeks. Our regiment was encamped for about a week at Algiers, opposite New Orleans, and it was very rainy weather during the time; in consequence, dysenteric affections became numerous. At the present time, the troops stationed at Santa Fe are suffering severely with these diseases, and it will not be denied that Santa Fe is a healthy town.

The water used by the troops at the Brazos, is obtained by digging small wells in the sand, usually to the depth of two feet. The water obtained from a well recently made was not very unpalatable, being the rain water contained in the upper surface of the sand, but in a short time the salt water from beneath would be mixed with it, thus rendering the well useless, so that new wells were constantly being made, and as the space occupied by the troops was only about half a mile in extent, and one hundred and fifty yards wide, (in rainy and stormy weather all the rest of the island being covered with water,) and as this sand hill ridge has been occupied by troops since the 20th of May last, even as many as three regiments have been stationed here at one time, it can readily be understood how the water of this ridge is affected.

The troops that have, from time to time, sojourned at the Brazos, have been for the most part volunteers, and they have had much more to learn than the drill and discipline. They have been compelled to take a few lessons in the culinary art—particularly so far as related to the cooking of pork and beans—a knowledge of which was not obtained until the pains of colic had been experienced more than once. It would be fair to say that the beans of every volunteer regiment are not half cooked, for, at least, the first month of service. Besides, the young soldier is apt to indulge in every excess. He will lie down on the wet ground without his blanket. The old soldier is more prudent—he may drink a little too much whiskey, (if he can get it,) but he will not expose himself unnecessarily to the sun's heat at mid-day, in fishing or hunting. Neither will he eat the coarse and unwholesome food that a recruit will swallow with avidity. The old soldiers of our regiment were the only men who would not indulge in eating red fish, oysters and crabs, whilst on the island. They were influenced, in part, by the example of the Mexicans, who eschew these luxuries during the summer months.

The country between Matamoras and the mouth of the Rio Grande, is low, with lakes every few miles, between which is interspersed the chapparel and prairie—the only elevation being the ridge of Burita, upon which the village is situated, and one nearly parallel with it on the opposite side of the river. These ridges, commencing at Burita, extend up the river about a mile. These elevations have been occupied by troops during the last summer, and I can speak for those encamped upon the ridge of Burita, as having enjoyed a very good share of health. There was a marked improvement in the health of the St. Louis Legion, after they encamped here. Red fish, oysters and crabs, could not now be obtained. Good water was within reach, and the beans were boiling in the camp kettles at an earlier hour than formerly. Here, too, was felt the delightful and invigorating sea-breeze, but sleep was not so sweet as at the Brazos. Centipedes, (some of them six inches in length,) tarantulas, and other venemous and creeping things, would travel over a man's nose, occasionally, and wake him up before reveille.

Immediately south of Burita, there is a fresh water lake of considerable size, and about half a mile on the opposite side of the ridge there is a salt lake. Fresh and salt lakes may be seen in close contiguity in this vicinity.

The Mexicans in Matamoras, and those who live at the ranches in the neighborhood, are as healthy a looking people as I ever saw. I visited, during the months of July and August last, many ranches, where I saw children, and I do not remember to have seen one child that had an unhealthy appearance. In many regions of this (Mississippi) valley, during the same months, it would not be surprising to find half the members of every family labouring under remittent and intermittent fevers. The only sick Mexican I saw whilst in the country, (except the wounded in the Hospital at Matamoras, (was a woman with intermittent fever, at Brazos Island. I was afterwards informed by an old Frenchman, who had lived for many years on a ranche near Matamoras, that the fever and ague was the only disease that prevailed in the neighbourhood, but that the "chills" were not as severe as those he used to have in Louisiana—here the patient got well in a few days, without, perhaps, being obliged to keep the bed.

I have remarked that the Mexicans have, universally, good teeth—an indication, certainly of good health, and I venture the assertion that there are as many old people, according to the population, as can be found in any part of the United States. I will further state, that in Matamoras, I became acquainted with several American merchants who had resided in that city for several years. They informed me that the country was healthy—that they had enjoyed better health in Mexico than in the United States. I therefore believe that the great mortality amongst our troops upon the Rio Grande, during the last summer was owing to the imprudence of the men—to bad cooking—to a neglect of proper police, in most of the volunteer regiments, and to the necessity which compelled the soldier to lie upon the wet ground during a wet season.

In order to show the number of diarrhœa cases, in comparison with all other diseases, I will here give an extract from my monthly report of "sick and wounded" for June. The regiment, during that month, was stationed at the Brazos Island.

| NAME OF DISEASE. | | | | | | | | | | | | | | | | | | | | REMAINING. | | | MEAN STRENGTH. | | |
|---|---|---|---|---|---|---|---|---|---|---|---|---|---|---|---|---|---|---|---|---|---|---|---|---|---|
| Diarrhœa. | Dysentery, | Remittent Fever, | Intermittent Fever, | Ulcers, | Catarrh, | Rheumatism, | Opthalmia, | Erysipelas, | Pleurisy, | Asthma, | Hernia Hæmoralis, | Gonorrhœa, | Syphilis, | Colic, | Wounds, | Total, | Returned to duty, | Discharged from service, | Dead, | Sick, | Convalescent, | Total, | Officers, | Men, | Total, |
| 170 | 9 | 36 | 18 | 7 | 7 | 6 | 2 | 7 | 4 | 2 | 1 | 2 | 3 | 8 | 4 | 286 | 229 | 12 | 1 | 38 | 19 | 57 | 30 | 601 | 601 |

Most of these cases of diarrhœa were preceded by colic, and could be traced to some imprudence in eating. The most successful mode of treatment I found, was to empty the bowels with castor oil, particularly when there was tenderness or pain over the region of the abdomen, and then to administer large doses of opium, 3 or 4 grains, at intervals of four hours, until the bowels were constipated, and after waiting forty-eight hours to give a dose of castor oil and laudanum. This was the only plan of treatment that was curative. Hyd. cum. cocta. dover's powder, with calomel, etc., were given without success at first. Though the cases of diarrhœa were so numerous, yet we did not lose a man of our regiment with that disease.

Most of the cases of remittent and intermittent fevers supervened upon diarrhœa. The remittent fevers were of a low form and very obstinate in their character. What retarded recovery, especially in these cases, was a despondent state into which almost every patient sank. After a man had suffered with fever for a week, he either made up his mind to die, or became so dejected that it was almost impossible to persuade him that he would recover, or to rouse his feelings in any way. I saw a few cases of pure nostalgia, and I believe there were many such, during the first six months of service, amongst the young men of the army. The marasmus, after remittent fever, was striking, and convalescence remarkably slow. These patients had the same cadaverous appearance and haggard expression of countenance as is common to children who are laboring under tabes mesenterica. There was also that loose and wrinkled condition of the skin of the abdomen which is common in such cases. Diffusible stimulants were very freely given in these cases, and with the most happy effects.

Two cases of wounds came under my notice that are worth mentioning, on account of the result. The first occurred in the Fifth Louisiana Regiment, (Col. Peyton's,) whilst stationed at Burita. Two men, previously good friends, had been drinking together, when an altercation ensued, and one of them drew a large Bowie knife and plunged it into the breast of the other. I reached the wounded man at the same moment with the surgeon of his regiment. On examination, we found a piece of the lung, two and a half, or three inches in length, protruding from the wound, which was about an inch below the left nipple—the knife passing between the ribs, downwards and outwards. The wound was at least three inches in length. After consultation, we concluded to introduce the wounded portion of lung within the thorax, and to close the external wound with the interrupted suture. The man was kept upon the most strict antiphlogistic treatment, and some twelve or fifteen days after, when I last saw him, there was every reason to believe that he would entirely recover. The knife with which this wound was given was two inches wide, and it must have penetrated the lung, four inches. On reflection I am not convinced that our practice in this case was the best that could have been adopted. The wounded portion of lung had only an attachment of three-fourths of an inch, and would it not have been better surgery to have clipped it off than to have replaced it within the cavity of the chest?

The other was a case of gun-shot wound, which occurred accidentally. A man was shot in the left axillæ, with a musket ball; the man who fired the gun being immediately opposite, and about one hundred and twenty yards distant. Being absent from camp I did not see the wounded man until the evening of the second day after the accident. On examination I could find no signs of the ball. The man was laboring under distressing dyspnœa. I learned that he had expectorated blood freely, when first shot, but now his cough was suppressed, and he could not expectorate at all. The left cavity of the chest seemed to be half full of blood, and on raising the man and turning him on the left side, at least half a pint of blood escaped through the wound. The following day he was bled twice, and the treatment was strictly antiphlogistic. About a week after, the track of the ball was plainly to be seen. After passing through the chest it made its exit half way, and just below the spine of the scapula; thence glancing inwards and downwards, lodged near the spinous process of the twelfth dorsal vertebræ, where I extirpated it. Beattie, the man whose wound I have just been describing, lives in this city. Prior to the accident he was a robust and healthy man, but he has now become thin and wan, and is frequently troubled with a cough.

*St. Louis Med. and Surg. Jour.*

